# A Multifunctional, Low-Volume Resuscitation Cocktail Improves Vital Organ Blood Flow and Hemostasis in a Pig Model of Polytrauma with Traumatic Brain Injury

**DOI:** 10.3390/jcm10235484

**Published:** 2021-11-23

**Authors:** Alexander E. St. John, Xu Wang, Kristyn Ringgold, Esther B. Lim, Diana Chien, Matthew L. Statz, Susan A. Stern, Nathan J. White

**Affiliations:** Department of Emergency Medicine, University of Washington School of Medicine, Seattle, WA 98195, USA; aestjohn@uw.edu (A.E.S.J.); xuwang58@uw.edu (X.W.); kringgo@uw.edu (K.R.); esther.br.lim@gmail.com (E.B.L.); chiend7@uw.edu (D.C.); matt.statz@gmail.com (M.L.S.); sstern@uw.edu (S.A.S.)

**Keywords:** multifunctional resuscitation fluid, damage control resuscitation, fibrinogen, hemostasis, perfusion

## Abstract

The resuscitation of polytrauma with hemorrhagic shock and traumatic brain injury (TBI) is a balance between permissive hypotension and maintaining vital organ perfusion. There is no current optimal solution. This study tested whether a multifunctional resuscitation cocktail supporting hemostasis and perfusion could mitigate blood loss while improving vital organ blood flow during prolonged limited resuscitation. Anesthetized Yorkshire swine were subjected to fluid percussion TBI, femur fracture, catheter hemorrhage, and aortic tear. Fluid resuscitation was started when lactate concentration reached 3–4 mmol/L. Animals were randomized to one of five groups. All groups received hydroxyethyl starch solution and vasopressin. Low- and high-dose fibrinogen (FBG) groups additionally received 100 and 200 mg/kg FBG, respectively. A third group received TXA and low-dose FBG. Two control groups received albumin, with one also including TXA. Animals were monitored for up to 6 h. Blood loss was decreased and vital organ blood flow was improved with low- and high-dose fibrinogen compared to albumin controls, but survival was not improved. There was no additional benefit of high- vs. low-dose FBG on blood loss or survival. TXA alone decreased blood loss but had no effect on survival, and combining TXA with FBG provided no additional benefit. Pooled analysis of all groups containing fibrinogen vs. albumin controls found improved survival, decreased blood loss, and improved vital organ blood flow with fibrinogen delivery. In conclusion, a low-volume resuscitation cocktail consisting of hydroxyethyl starch, vasopressin, and fibrinogen concentrate improved outcomes compare to controls during limited resuscitation of polytrauma.

## 1. Introduction

The leading causes of death from trauma are traumatic brain injury (TBI) and hemorrhage [1,2]. The goals of resuscitation for TBI and hemorrhagic shock (HS) are seemingly at odds during prolonged damage control resuscitation (pDCR). HS is best managed with limited-volume resuscitation to minimize blood loss and promote hemostasis [3]. Conversely, TBI worsens with hypotension in a time- and dose-dependent manner [4]. This challenge is substantiated by the finding that trauma patients with combined HS and TBI suffer higher mortality and worsened coagulopathy than those with HS alone [5]. Current strategies employed in the United States military’s Tactical Combat Casualty Care (TCCC) guidelines include administration of fluid to maintain a palpable radial pulse, which falls on the side of permissive hypotension and limited resuscitation, potentially allowing a second-hit to vital organ perfusion, including the injured brain [6].

The common goal behind management of HS and TBI is supporting vital organ perfusion, but HS and TBI pose differing barriers to this. HS is caused by intravascular volume loss, while TBI impairs systemic perfusion through neurovascular dysregulation, leading to decreased cardiac output and vascular tone [7,8,9]. Both HS and TBI are also associated with coagulopathy, which contributes to worsened blood loss [10,11]. An ideal resuscitation would support hemostasis to limit hemorrhage, increase cardiac output, and maintain vascular tone to optimize perfusion. If this could be achieved in a low-volume fluid, its portability could make it available very early after injury, including in far-forward combat scenarios and prolonged extrication civilian scenarios.

We previously showed that hydroxyethyl starch (HES), used as the first-line resuscitation fluid by the United States military, in combination with vasopressin improved vascular tone during resuscitation of HS [12]. This combination increased mean arterial blood pressure and vital organ perfusion. However, it came at a cost of increased hemorrhage volumes, highlighting the need to also address hemostasis directly when formulating resuscitation strategies.

Fibrinogen plays a critical role in hemostasis after injury because low fibrinogen levels are associated with increased mortality in trauma patients, and it is the first coagulation factor to reach a critically low level after trauma [13,14,15]. Fibrinogen supplementation has demonstrated benefit by decreasing blood loss and improving survival in experimental models of both solid organ and arterial hemorrhage [16,17].

TXA is a synthetic lysine analog that competitively binds the functional site of plasminogen, effectively blocking the lysis of fibrin and fibrinogen [18,19]. It has been shown to decrease mortality when given early to trauma patients with suspicion of active hemorrhage [20,21]. TXA is highly water soluble, enabling it to be delivered as a relatively low-volume solution, which makes it well suited for use in a low-volume resuscitation cocktail. Additionally, its ability to inhibit fibrinogenolysis in addition to fibrinolysis would mitigate the loss of administered fibrinogen in a blood environment that is potentially highly proteolytic.

Here, we use a large animal model of polytrauma with TBI and free internal bleeding to test the hypothesis that limited infusion of a multifunctional resuscitation cocktail supplemented with fibrinogen improves outcomes through support of the hemodynamic and hemostatic response during prolonged limited resuscitation.

## 2. Materials and Methods

**Figure 1:**
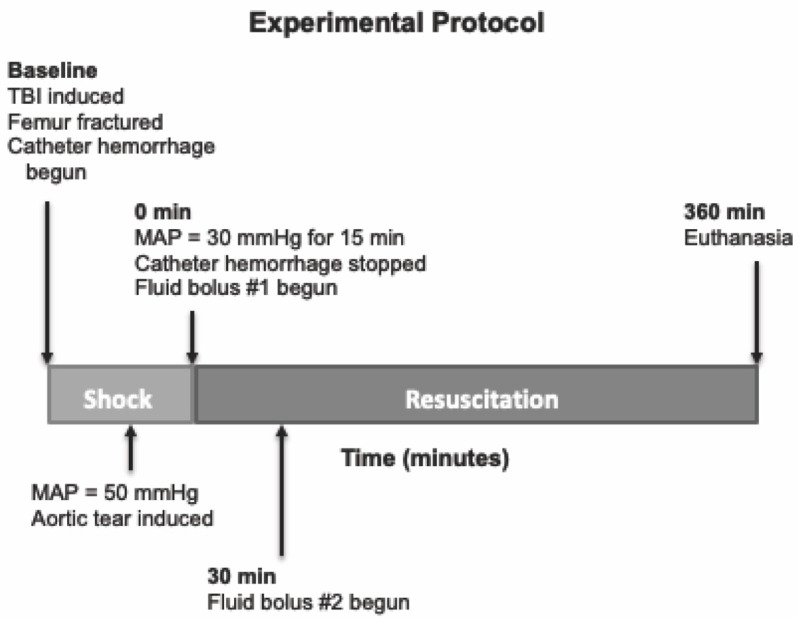
Overview of experimental protocol timeline. TBI, traumatic brain injury. MAP, mean arterial pressure.

We tested a multifunctional, low-volume resuscitation cocktail in a swine model of severe polytrauma that includes TBI and hemorrhagic shock. This cocktail was meant to be compatible with resuscitation early after injury, even in austere environments. An overview of the experimental protocol timeline is provided in Figure 1. The experimental protocol was approved by the University of Washington Office of Animal Welfare.

### 2.1. Animal Preparation and Instrumentation

A total of 34 immature (25–30 kg) female Yorkshire swine (*Sus scrofa domestica*) were acclimated to the animal facility for 3–5 days with free access to food and water and were fasted overnight prior to study. On the morning of this study, animals were sedated with ketamine (30 mg/kg intramuscularly), and anesthesia was induced with isoflurane (2–4%) via nasal cone. Upon reaching a surgical plane of anesthesia, animals were intubated, and the isoflurane concentration was reduced to 1–2%. FiO_2_ was titrated to an arterial O_2_ saturation > 95%. End-tidal CO_2_ (ETCO_2_) was monitored continuously and maintained at 35–40 torr via adjustment of ventilator rate and tidal volume. Buprenorphine (0.01 mg/kg IM) was administered for analgesia. Animals were placed on a warming blanket, and body temperature was monitored via pulmonary artery catheter.

After anesthesia induction, the right side of the neck, both femoral areas, and the anterior abdominal wall were shaved and widely prepared with povidone-iodine. Animals were instrumented with ECG leads, an ear pulse oximeter, and a Foley catheter to monitor urine output. Right femoral artery and venous catheters were placed for blood sampling and fluid/drug administration. A right carotid artery introducer catheter was placed for continuous BP monitoring and blood sampling. A 5-French pig-tail catheter was placed via the right carotid into the left ventricle for pressure monitoring and colored microsphere injection for regional blood flow measurements. A pulmonary artery thermodilution catheter was inserted via the right external jugular vein and advanced into the pulmonary artery for central venous pressure (CVP), mean pulmonary artery pressure (MPAP), cardiac output (CO), and core temperature monitoring, as well as mixed venous blood (MVO_2_) sampling.

Following catheter placement, a laparotomy was performed via midline abdominal incision for splenectomy and placement of an infrarenal aortotomy for creation of an aortic tear. A midline 10 cm longitudinal abdominal incision was made, and the spleen was isolated and removed. Following splenectomy, the retroperitoneal fascia was incised, and the ventral surface of the infrarenal aorta was exposed. A 4.0 monofilament stainless steel surgical wire was placed through the ventral wall of the infrarenal aorta into the aortic lumen, advanced, and exited at a point on the aorta 4 mm distal from insertion. The wire ends were then exteriorized through the abdominal incision, and the incision was closed with surgical staples. While supine, a 10 cm incision was made over the right anterior mid-femur. The anterolateral surface of the mid-femur was exposed by blunt dissection for subsequent fracture.

Next, pigs were placed in the prone position with their head placed in a stabilizer. The scalp was widely prepared with povidone iodine. A circumferential incision was made, the scalp reflected posteriorly, and the cranium exposed. A 16 mm-diameter craniotomy was performed in the right parietal region adjacent to the sagittal suture and anterior to the coronal suture. A T-shaped bolt was screwed into the craniotomy so it abutted the intact dura. This bolt was connected to the fluid percussion device. Another craniotomy (5 mm) was performed in the left posterior parietal region, and a neonatal intraventricular catheter (Phoenix Biomedical Corp., Bolton, ON, Canada) was placed in the left lateral ventricle and connected to an intracranial pressure (ICP) monitor. A brain temperature-monitoring probe was also placed in this same craniotomy site. A third craniotomy site (5 mm) was prepared just anterior to the inion for placement of a sagittal sinus catheter for cerebral venous blood sampling. Finally, a fourth craniotomy (3 mm) was made in the left frontoparietal region for insertion of a Licox tissue O_2_-sensing probe. All craniotomy sites were sealed with dental cement.

### 2.2. Injury Protocol

Thirty minutes after instrumentation, baseline metabolic, hemodynamic, and coagulation measurements were made. Next, animals underwent TBI by fluid percussion using a device consisting of a saline-filled tube and a weighted pendulum. The saline-filled tube is connected directly to the bolt installed on the animal’s skull. To induce injury, the pendulum was pulled back a standardized distance and allowed to fall, striking a rubber seal on the end of a plexi-glass piston. The resulting fluid wave that is generated in this closed system transmits a 15 msec pressure pulse of 3–3.5 atm to the intact dura. A high-pressure transducer is attached to the craniotomy bolt (directly above the site of injury), which permits quantification of the delivered pressure. This percussion injury has been shown in previous models to result in moderate TBI [22]. After TBI, the animal is immediately placed in the supine position. At this point, femur fracture was induced by firing a captive bolt pistol (Schermer Stunner Model MKL, Karl Schermer and Co., Karlsruhe, Germany) directly against the exposed anterolateral surface of the right femur, as has been described in previous study [23]. This injury creates an open, displaced, comminuted mid-shaft femur fracture with surrounding soft tissue injury. Simultaneously, catheter hemorrhage was begun via the left femoral arterial catheter. Hemorrhage was controlled by computer-driven roller pump with an exponentially decreasing rate over time to mimic the kinetics of free hemorrhage, as has been previously described [24,25,26]. When mean arterial pressure (MAP) reached 50 mmHg, aortic tear was induced by pulling the exteriorized wire loop, allowing free intraperitoneal hemorrhage. After aortic tear, animals were maintained at a MAP of 30 mmHg by toggling catheter hemorrhage on or off. During this period of shock, serial lactic acid measurements were taken to monitor severity of shock. Upon reaching the target lactate concentration of 3–4 mmol/L, which usually occurred after approximately 15 min, resuscitation was begun. A similar injury protocol including the fluid percussion TBI, initial catheter hemorrhage, aortic tear at MAP of 50 mmHg, and maintenance of MAP at 30 mmHg to induce severe shock has been used previously [24,25,26]. The model did not include femur fracture, but it resulted in a 1 h mortality near 90% with no resuscitation that was modifiable with varying qualities of resuscitation.

### 2.3. Resuscitation Protocol

Resuscitation consisted of one of five randomly assigned resuscitation fluids divided into two boluses given 30 min apart, in keeping with TCCC guidelines at the time of experiment design. The fluids were as follows:

- Albumin control (AC): Hextend 14 mL/kg, 0.4 U/kg vasopressin, and 100 mg/kg albumin (chosen to equal the same molar concentration of high-dose fibrinogen below) (*n* = 5)
- Low-dose fibrinogen (LF): Hextend 14 mL/kg, 0.4 U/kg vasopressin, and 100 mg/kg fibrinogen concentrate (*n* = 8)
- High-dose fibrinogen (HF): Hextend 14 mL/kg, 0.4 U/kg vasopressin, and 200 mg/kg fibrinogen concentrate (*n* = 8)
- Albumin + tranexamic acid control (TA): Hextend 14 mL/kg, 0.4 U/kg vasopressin, 100 mg/kg albumin, and 15 mg/kg TXA (*n* = 5)
- Tranexamic acid + low-dose fibrinogen (TLF): Hextend 14 mL/kg, 0.4 U/kg vasopressin, 100 mg/kg fibrinogen concentrate, and 15 mg/kg TXA (*n* = 8)

At the initiation of resuscitation (“R0”), the first bolus was infused over 10 min. The second bolus was begun 30 min after the onset of resuscitation (“R30”) and was given at the same rate. After completion of the second bolus, no further resuscitation was given.

Animals were monitored for up to 6 h after beginning of resuscitation (“R360”) or until time of death, at which time animals were euthanized with an overdose of pentobarbital (100 mg/kg). Death was defined as loss of pulse pressure on arterial waveform.

### 2.4. Outcome Measurements

There were two primary outcome measurements: survival to 6 h and intraperitoneal hemorrhage volume. Secondary outcome measures included systemic metabolic resuscitation (lactate, pH, base deficit), hemodynamics (MAP and cardiac output by thermal dilution), cerebral-specific resuscitation (cerebral perfusion pressure, intracranial pressure, sagittal sinus venous hemoglobin oxygen saturation, and cerebral lactate), and organ-specific blood flow measurements by color-tagged microspheres.

Intraperitoneal hemorrhage was measured at 6 h or time of death by opening the abdomen and collecting shed blood using pre-weighed laparotomy sponges. Systolic, diastolic, and mean arterial pressure, respiratory rate, temperature, and ETCO_2_ were recorded at baseline and continuously throughout the protocol. ECG was monitored continuously for HR and arrhythmias. Cardiac output was measured via the thermodilution technique at baseline and every 15 min to R120 and every 30 min thereafter. ICP was monitored throughout the protocol. Cerebral venous O_2_ saturation was measured at baseline, start of resuscitation, 15 min intervals to R120, 30 min intervals to R240, and 1 h intervals thereafter. Cerebral perfusion pressure, cerebral O_2_ delivery, cerebral O_2_ extraction ratio, and cerebral metabolic rate of O_2_ were calculated from the above parameters. Microspheres (Dye-Trak Microspheres, Triton Inc., Seattle, WA, USA; intravenous—1 mL, 3 million/mL) were purchased in sterile vials and reconstituted in sterile saline on the day of the experiment for measurement of cerebral, cardiac, renal, and intestinal blood flow. Microspheres were injected into the left ventricle via catheter at baseline and at R30, R90, R180, and R360 and organ-specific blood flow was determined using the reference sample method. Blood was collected at baseline, at the start of resuscitation, and at 15 min intervals until R120, at 30 min intervals until R240, and at 1 h intervals thereafter for hemoglobin, hematocrit, arterial, mixed venous, and sagittal sinus blood gas measurements, and arterial and sagittal sinus lactate measurements (Radiometer Medical: ABL 505, EML 100, and OSM3, Copenhagen, Denmark). White blood cells, platelets, prothrombin time (PT), partial thromboplastin time (PTT), fibrinogen, rotational thromboelastometry (ROTEM, Instrumentation Laboratory, Bedford, MA, USA) with whole blood and fibrin-specific clot maximal amplitude measurements using platelet poor plasma obtained by centrifugation were measured at baseline, and at R0, R60, R120, R240, and R360.

### 2.5. Statistical Analysis

Cross-sectional data were compared at a single point in time or aggregated over time with repeated measure two-way analysis of variance (ANOVA) testing the effects of protocol time and study group with interaction and *χ*^2^ tests to compare continuous and categorical variables, respectively, between groups. Tukey adjustment was made for multiple comparisons. An overall *p* value of less than 0.05 was considered statistically significant for all analyses. Survival was compared between groups using time-to-event Kaplan–Meier analysis. To test the effect of including fibrinogen, the AC group was compared to LF and HF. To test the effect of fibrinogen dose, LF was compared to HF. To test the effect of TXA in the absence of fibrinogen supplementation, AC was compared to TA. To test the effect of TXA in addition to fibrinogen supplementation, LF was compared to TLF. The low-dose fibrinogen group was chosen for TXA supplementation, because we found it had slightly lower hemorrhage volumes than the high-dose fibrinogen group. Finally, to test for an overall effect of fibrinogen supplementation, all groups containing fibrinogen concentrate were pooled and compared to all pooled control groups. All statistical analysis was performed with SAS JMP v.12 (SAS, Cary, NC, USA).

Preliminary work using this polytrauma model found a 0% 6 h survival without fibrinogen concentrate included in the cocktail. Therefore, in order to detect a clinically relevant 50% increase in survival with 80% power while assuming a null 30% difference, we would require at least 5 animals in each experimental vs. control group and at least 8 animals per group when comparing fibrinogen and TXA dosages, assuming a 5% null difference to be relevant with alpha = 0.05. Therefore, non-fibrinogen control groups were allotted 5 animals and fibrinogen- and TXA-containing groups were allotted 8 animals each. This number of animals in each group also allowed us to detect a difference in lactate concentration of at least 2 mmol/L, a difference in cerebral perfusion pressure of 10 mmHg, and a change in intraperitoneal blood loss of 5 mL/kg in up to 4 experimental groups with 80% power.

## 3. Results

### 3.1. Effect of Fibrinogen on Survival and Blood Loss

**Figure 2:**
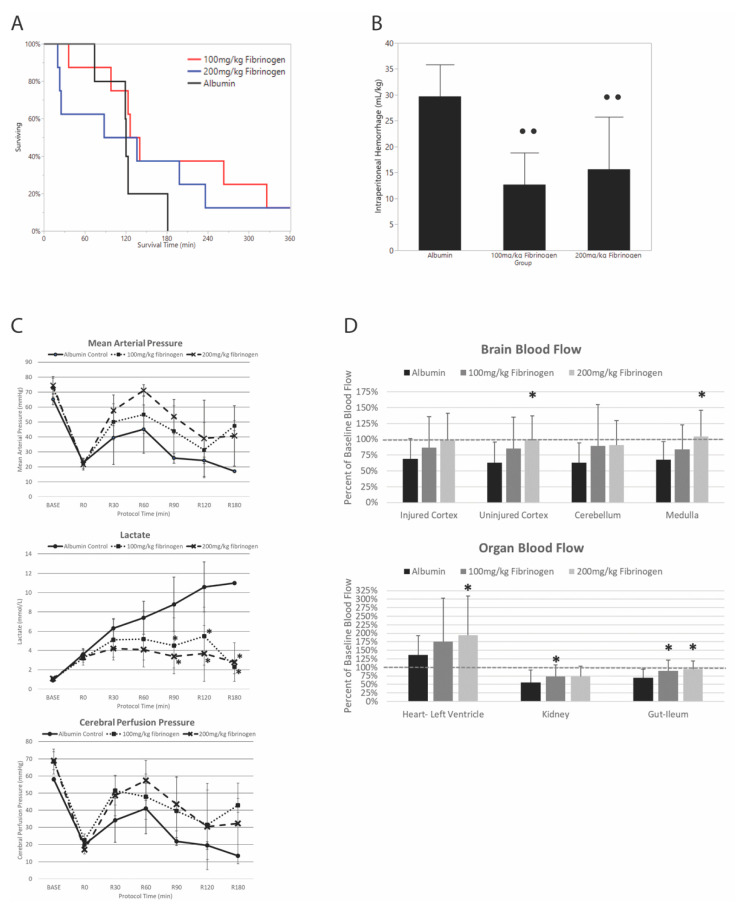
Effect of low- and high-dose fibrinogen concentrate on survival time (**A**), intraperitoneal blood loss (**B**), hemodynamics and lactate (**C**), and vital organ blood flow (**D**), compared to albumin resuscitation. All groups received Hextend and vasopressin. Error bars represent standard deviation. • • and *, *p* < 0.05 compared to albumin with Tukey HSD adjustment for multiple comparisons.

No animals survived to 6 h in the albumin group (0/5), while 13% of animals (1/8) survived to 6 h in each fibrinogen group (Figure 2A). There were no differences in overall survival between groups (Kaplan–Meier log rank *p* = 0.4). Intraperitoneal blood loss was significantly decreased with LF and HF compared to AC. There was no difference in intraperitoneal blood loss when comparing LF to HF (*p* = 0.7) (Figure 2B).

### 3.2. Effect of Fibrinogen on Hemodynamics and Resuscitation

There was a significant effect of study group on mean arterial pressure during resuscitation (ANOVA group effect *p* < 0.001), but no interaction with the time variable. Overall mean (SD) MAP during resuscitation was 37.7 (18.4) mmHg with albumin, which was significantly lower than both low-dose fibrinogen at 46.3 (19.9) mmHg (*p* = 0.002) and high-dose fibrinogen 50.8 (22.7) mmHg (*p* < 0.001). There were no individual differences between group MAPs at single time points.

There was a significant group effect and interaction on lactate concentration (ANOVA group effect *p* < 0.001, interaction effect *p* < 0.001). Both fibrinogen-containing groups had significantly lower lactate concentrations compared to the albumin group at 90, 120, and 180 min of resuscitation (Figure 2C). There was no difference between fibrinogen-containing groups.

There was also a significant group effect of fibrinogen on cerebral perfusion pressure (CPP) during resuscitation (ANOVA group effect *p* = 0.006), without a significant interaction. Overall mean (SD) CPP during resuscitation was 31.7 (16.1) mmHg with albumin, which was significantly lower than both low-dose fibrinogen at 45.8 (19.5) mmHg (*p* = 0.001) and high-dose fibrinogen at 43.8 (22.3) mmHg (*p* = 0.002). There were no individual differences between group CPPs at single time points.

### 3.3. Effect of Fibrinogen on Brain and Vital Organ Blood Flow

Brain and vital organ blood flow was available for all groups up to and including the first 60 min of resuscitation and is reported as the percent of baseline blood flow for each organ measured prior to TBI and HS. MAP was too low after 60 min in the albumin control group to obtain accurate vital organ blood flow measurements. Measurements from the left and right kidneys compared and found to be not significantly different, so they were combined. There was a significant effect of treatment group on brain blood flow in some but not all of the various brain regions measured (Figure 2D). In the injured cerebral cortex and cerebellum, there was no effect of fibrinogen on blood flow during resuscitation (ANOVA group effect *p* values > 0.14). However, there was a significant increase in regional blood flow in the uninjured cortex and medulla with high-dose fibrinogen, compared to albumin control (ANOVA group effect *p* values < 0.033). Overall, most brain regions tended to increase in groups that received fibrinogen, with the high-dose fibrinogen group recovering nearly 100% of baseline flow during the first 60 min of resuscitation. Blood flow also tended to increase with fibrinogen in other vital organs including the heart, kidney, and gut. Left ventricular blood flow typically increased above baseline values during shock, and this response was significantly more pronounced in fibrinogen-containing groups (ANOVA group effect *p* = 0.04). There was also a significant effect of study group on kidney and ileum blood flow (ANOVA group effect *p* values < 0.04). The high-dose fibrinogen group had significantly increased blood flow to the left ventricle of the heart and ileum compared to albumin, and the low-dose fibrinogen group had significantly increased blood flow in the kidney and ileum compared to albumin controls.

### 3.4. Effects of Tranexamic Acid on Survival and Blood Loss

**Figure 3:**
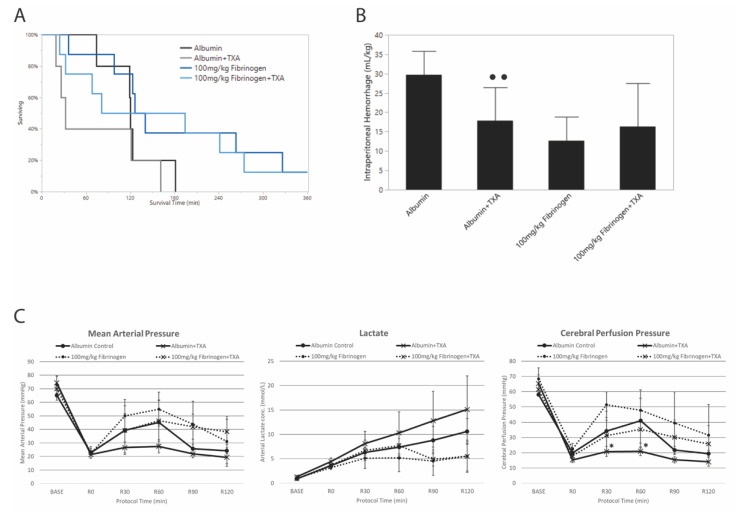
Effect of TXA on survival time (**A**), intraperitoneal blood loss (**B**), and hemodynamics and lactate (**C**). All groups received Hextend and vasopressin. Error bars represent standard deviation. • • and *, *p* < 0.05 compared to control group without TXA with Tukey HSD adjustment for multiple comparisons.

None of the animals in the AC or ATC groups survived to 6 h (0% survival). There was no difference in overall survival when comparing albumin alone to albumin+TXA (Kaplan–Meier log rank *p* = 0.3). One animal in each of the LF and TLF groups survived to 6 h (13% survival) and no statistical survival difference when comparing fibrinogen alone to fibrinogen+TXA (Kaplan–Meier log rank *p* = 0.7) (Figure 3A). Blood loss was significantly less when TXA was added to albumin, but this was not associated with increased survival. There was no difference in blood loss when TXA was added to fibrinogen concentrate (Figure 3B).

ROTEM data showed similarly low levels of fibrinolysis in all groups, regardless of whether TXA was administered (Table S1).

### 3.5. Effects of Tranexamic Acid on Hemodynamics and Resuscitation

Data were available for all groups only during the first 120 min of resuscitation due to deaths in the albumin+TXA group. (Figure 3C) When comparing AC to ATC, there was a lower MAP in the ATC group that did not reach significance (ANOVA interaction effect *p* = 0.058). There was a significant group effect on lactate (ANOVA group effect *p* = 0.003) with a significantly higher average (SD) lactate for ATC at 7.3 (5.4) mmol/L than with AC at 5.9 (3.5) mmol/L. There was also a significant interaction effect on CPP (ANOVA interaction *p* = 0.015) where adding TXA to albumin decreased CPP significantly at 30 and 60 min of resuscitation compared to albumin alone (Figure 3C).

There were no effects of TXA on MAP or lactate when tested in the presence of 100 mg/kg fibrinogen. Mean (SD) CPP was significantly decreased during the first 120 min of resuscitation with fibrinogen+TXA at 35.4 (17.4) mmHg compared to fibrinogen alone at 50.0 (20.2) mmHg (ANOVA group effect *p* = 0.001).

### 3.6. Effects of Tranexamic Acid on Organ Blood Flow

The addition of TXA to albumin decreased brain blood flow during the first 60 min of resuscitation in all regions of the brain sampled compared to albumin alone (ANOVA group effect *p* values ≤ 0.018), except for the uninjured cortex, where there was no effect. There was also no effect of TXA on organ blood flow (heart, kidney, gut) compared to albumin alone. There were no effects of TXA on vital organ blood flow when TXA was added to low-dose fibrinogen in comparison to low-dose fibrinogen alone, except for the ileum, where the 60-min blood flow measurement was significantly decreased with fibrinogen+TXA (57%) compared to fibrinogen alone (103%) (ANOVA interaction effect *p* = 0.04).

### 3.7. Pooled Effects of Fibrinogen

To test for an overall effect of fibrinogen regardless of its concentration or the presence of TXA, animals were pooled into two separate groups segregated by the presence or absence of fibrinogen in the resuscitation fluid. The fibrinogen group consisted of the 100 mg/kg fibrinogen, 200 mg/kg fibrinogen, and 100 mg/kg fibrinogen+TXA groups (*n* = 24 total). The no fibrinogen group consisted of the albumin, and albumin+TXA groups (*n* = 10 total). All animals received the same amount of Hextend and vasopressin during resuscitation.

**Figure 4:**
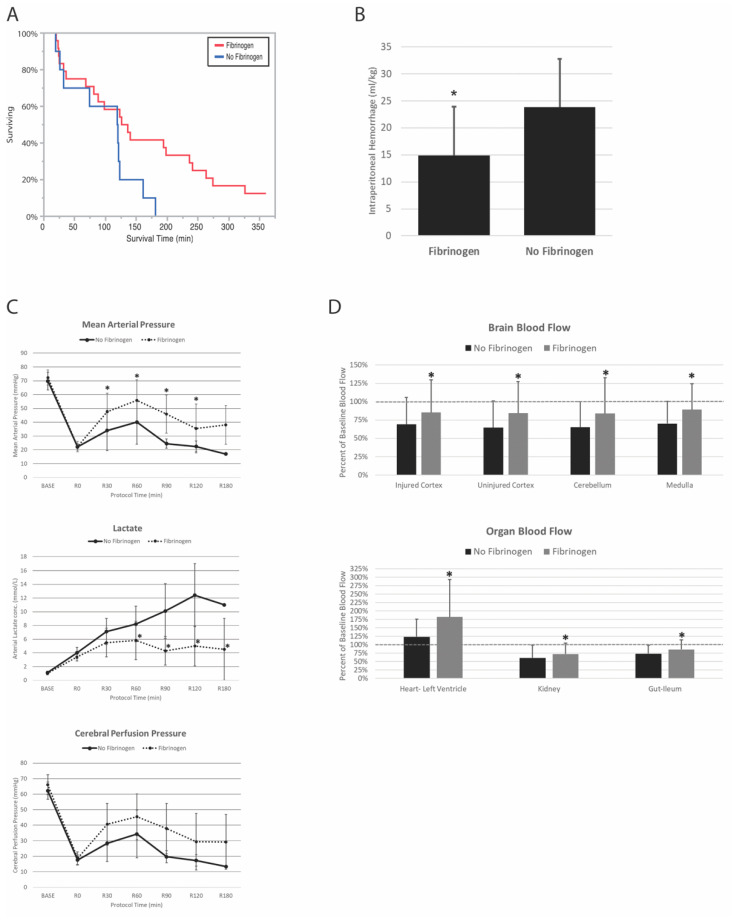
Combined effect of fibrinogen on survival time (**A**), intraperitoneal blood loss (**B**), hemodynamics and lactate (**C**), and vital organ blood flow (**D**). All animals received Hextend and vasopressin. Error bars represent standard deviation. * *p* < 0.05 compared to no fibrinogen with Tukey HSD adjustment for multiple comparisons.

Survival was significantly greater in the fibrinogen group (12.5%) vs. the no fibrinogen group (0%) (Kaplan–Meier log rank *p* = 0.04) (Figure 4A). Internal blood loss was also significantly less for the fibrinogen group (Figure 4B).

### 3.8. Pooled Effect of Fibrinogen on Hemodynamics and Resuscitation

There was a significant effect of fibrinogen on MAP measurable up to 240 min (ANOVA interaction effect *p* = 0.03), cerebral perfusion pressure (CPP) up to 240 min (ANOVA treatment effect *p* < 0.001), and blood lactate concentration measurable up to 180 min (ANOVA interaction effect *p* = 0.004). There was a significant interaction effect of study group for MAP and lactate concentration (ANOVA interaction effect *p* values < 0.024) (Figure 4C). MAP was significantly increased in the fibrinogen group at 30, 60, 90, and 120 min of resuscitation. Lactate was significantly decreased with fibrinogen from 60–180 min of resuscitation. Overall average (SD) CPP was significantly increased with fibrinogen at 40.5 (20.2) mmHg, compared to no fibrinogen at 30.9 (18.8) mmHg, but did not differ at individual time points.

Pooled analysis of the effect of fibrinogen on organ blood flow revealed that there was a significant overall effect of fibrinogen where blood flow was increased in all regions of the brain and all organs measured (all ANOVA group effects *p* ≤ 0.038) (Figure 4D). Individual differences included a significantly increased blood flow within the medulla, left ventricle, and kidney with fibrinogen at 60 min of resuscitation (ANOVA interaction term *p* values < 0.034).

## 4. Discussion

Our study has several important findings. First, the addition of fibrinogen concentrates to a low-volume resuscitation cocktail containing HES and vasopressin can improve survival and reduce blood loss during limited-volume resuscitation of polytrauma with TBI and noncompressible arterial hemorrhage. While the comparisons between the individual fibrinogen arms and their corresponding controls were not statistically significant, a post hoc pooled analysis of all of the fibrinogen arms compared to the non-fibrinogen arms showed a survival benefit. This effect appears to be related to improved hemostasis, rather than oncotic effects, as reflected by a decrease in hemorrhage volume. The hemostatic benefit of fibrinogen administration is further supported by the comparisons of fibrinogen-containing arms to the albumin controls, which showed that fibrinogen decreased blood loss, even in the setting of greater MAPs, which is a primary goal of damage control resuscitation in the setting of TBI.

The decreased hemorrhage volume seen in the pooled analysis of fibrinogen-containing arms was also accompanied by improved resuscitation quality. This was broadly reflected in better hemodynamic profiles, decreased lactate concentration, and improved vital organ blood flow, including to the heart and injured brain. These findings were corroborated by similar results in the individual comparisons between fibrinogen-containing arms and controls.

These findings are in keeping with prior human and animal studies showing that fibrinogen supplementation improves coagulation profile and hemostasis [16,17,27,28,29,30]. Our model shows its effectiveness in severe polytrauma that is meant to represent the most drastic battlefield injuries with limited access to volume resuscitation or surgical repair for several hours. There are multiple ongoing clinical trials to directly test the effect of early fibrinogen supplementation in humans after injury.

The threshold effect seen in our study with fibrinogen dosing is also consistent with existing literature. Two prior pig studies of resuscitation after trauma have shown a benefit to the administration of an amount of fibrinogen similar to our low-dose group with no additional benefit seen with higher doses [16,30]. The threshold amount ranges between 75 and 150 mg/kg. At higher concentrations, the oncotic effects of additional fibrinogen could counteract any marginal hemostatic benefit. This is consistent with the slightly higher blood pressures and hemorrhage volumes seen in our high- vs. low-dose fibrinogen groups.

Our model was limited in its ability to detect the impact of TXA. None of the experimental arms resulted in any significant fibrinolysis, as detected by ROTEM analysis. Because of this, none of the TXA-containing arms had any added benefit over their controls. Pigs tend to be hypercoagulable and less prone to hyperfibrinolysis after trauma than humans. However, this clearly is not representative of the human trauma population, which exhibits a complex and heterogeneous impact on fibrinolysis with well-demonstrated benefits of TXA to certain populations of trauma patients [20,21,31,32,33,34]. Therefore, interpretation of the impact of TXA on a low-volume resuscitation cocktail such as the one presented here should be suspended until further testing can be done in an appropriate model of fibrinolysis.

Sheppard et al. stated that the ideal resuscitation fluid would be universally infusible and easily portable, and it would provide both metabolic resuscitation and hemostatic support [35]. Traditional resuscitation fluids and donor blood products satisfy only one or two of these requirements, leaving a gap with major implications for both military and civilian prehospital care. Our findings represent important steps in the development of a more complete resuscitation fluid for these purposes. A low-volume resuscitation cocktail containing Hextend, vasopressin, fibrinogen, and possibly TXA shows benefits in all of the required areas, making it a promising candidate for further development toward prehospital use. Since it is now known that Hextend can contribute to coagulopathy and is no longer the preferred primary resuscitation fluid in TCCC guidelines, it is possible eliminating Hextend and incorporating blood transfusion would further improve the performance of this cocktail.

In conclusion, the addition of fibrinogen to a low-volume resuscitation cocktail containing HES and vasopressin improved survival, blood loss, and vital organ blood flow. In conjunction with our previous findings regarding the benefits of vasopressin, we have created a multifunctional resuscitation cocktail that is amenable to use by military medics in far-forward settings with severe polytrauma patients. Further study should focus on testing for possible additional benefit with adding TXA in an appropriately fibrinolytic model to further optimize the cocktail for human use and possibly in a model that incorporates TCCC-recommended fresh whole blood transfusion.

## Data Availability

The data presented in this study are openly available in FigShare (doi:10.6084/m9.figshare.16755676).

## Supplementary Materials

The following are available online at https://www.mdpi.com/article/10.3390/jcm10235484/s1, Table S1: Rotational thromboelastometry (ROTEM) data.
