# Supplementary material for: A Multifunctional, Low-Volume Resuscitation Cocktail Improves Vital Organ Blood Flow and Hemostasis in a Pig Model of Polytrauma with Traumatic Brain Injury
Source: J Clin Med. 2021 Nov 23;10(23):5484. doi: 10.3390/jcm10235484 (PMC8658540; doi:10.3390/jcm10235484)
Supplement: Supplementary file 1 [file jcm-10-05484-s001.zip › jcm-1432039-supplementary.pdf]

Supplementary Table S1. Rotational thromboelastometry (ROTEM) data. Data are presented as mean (standard deviation). WB, whole blood; MCF, maximal clot firmness; LI30, lysis index at 30 min.

		Protocol Time					
**Treatment Group**	**ROTEM Parameter**	**Baseline**	**R0**	**R60**	**R120**	**R240**	**R360**
Albumin Control	WB EXTEM MCF	74.7 (5.5)	71.7 (4.5)	60.5 (10.6)	58.0 (5.7)	-	-
	Plasma EXTEM MCF	36.7 (2.5)	30.0 (1.4)	11.7 (4.2)	11.7 (4.2)	-	-
	Plasma APTEM MCF	35.3 (3.1)	28.0 (1.4)	10.0 (5.6)	9.3 (3.2)	-	-
	Plasma EXTEM LI30	100.0 (0)	100.0 (0)	99.5 (0.7)	100.0 (0)	-	-
	Plasma APTEM LI30	100.0 (0)	100.0 (0)	100.0 (0)	100.0 (0)	-	-
Low-Dose Fibrinogen	WB EXTEM MCF	76.5 (1.3)	76.4 (2.1)	69.3 (2.2)	70.8 (1.7)	71.0 (2.0)	71.0 (0)
	Plasma EXTEM MCF	42.3 (3.8)	36.0 (6.7)	22.4 (4.6)	20.7 (5.3)	24.7 (5.5)	24.0 (2.8)
	Plasma APTEM MCF	41.3 (3.3)	37.3 (4.6)	22.7 (3.4)	20.2 (3.7)	24.7 (6.4)	25.0 (1.4)
	Plasma EXTEM LI30	100.0 (0)	100.0 (0)	100.0 (0)	100.0 (0)	100.0 (0)	100.0 (0)
	Plasma APTEM LI30	99.9 (0.4)	100.0 (0)	100.0 (0)	100.0 (0)	100.0 (0)	100.0 (0)
High-Dose Fibrinogen	WB EXTEM MCF	75.8 (3.1)	74.8 (2.3)	72.6 (3.0)	75.3 (1.5)	77.5 (0.7)	80.0 (0)
	Plasma EXTEM MCF	43.0 (16.8)	40.6 (15.7)	25.8 (4.9)	30.0 (7.1)	31.0 (1.4)	29.0 (0)
	Plasma APTEM MCF	42.1 (17.5)	41.0 (17.2)	25.8 (3.6)	29.3 (5.6)	30.5 (0.7)	31.0 (0)

	Plasma EXTEM LI30	99.6 (1.1)	99.7 (0.5)	99.8 (0.4)	99.8 (0.5)	100.0 (0)	100.0 (0)
	Plasma APTEM LI30	99.4 (0.8)	99.7 (0.5)	100.0 (0)	100.0 (0)	100.0 (0)	100.0 (0)
Albumin + TXA	WB EXTEM MCF	72.8 (7.5)	73.0 (6.7)	56.5 (14.9)	59.0 (14.1)	-	-
	Plasma EXTEM MCF	35.4 (7.7)	32.4 (6.4)	11.0 (5.7)	10.0 (4.2)	-	-
	Plasma APTEM MCF	35.6 (7.4)	31.2 (6.1)	8.5 (3.5)	7.5 (5.0)	-	-
	Plasma EXTEM LI30	99.8 (0.5)	100.0 (0)	100.0 (0)	100.0 (0)	-	-
	Plasma APTEM LI30	99.8 (0.5)	100.0 (0)	100.0 (0)	89.5 (14.9)	-	-
Low-Dose Fibrinogen + TXA	WB EXTEM MCF	76.4 (3.3)	76.0 (4.0)	69.2 (4.5)	67.5 (3.0)	70.0 (4.0)	71.0 (0)
	Plasma EXTEM MCF	40.1 (3.4)	35.9 (5.8)	17.8 (3.8)	19.3 (5.8)	22.7 (5.5)	19.0 (0)
	Plasma APTEM MCF	39.4 (3.5)	35.5 (7.1)	19.0 (2.6)	17.8 (2.9)	22.7 (3.5)	20.0 (0)
	Plasma EXTEM LI30	99.9 (0.4)	99.9 (0.4)	100.0 (0)	100.0 (0)	99.3 (1.2)	100.0 (0)
	Plasma APTEM LI30	99.9 (0.4)	99.9 (0.4)	99.2 (1.0)	97.8 (4.5)	99.0 (1.8)	100.0 (0)
